# Unweaving the Cognitive Map: A Personal History

**DOI:** 10.1002/hipo.23674

**Published:** 2024-12-19

**Authors:** Kate J. Jeffery

**Affiliations:** ^1^ School of Psychology & Neuroscience University of Glasgow Glasgow UK

**Keywords:** configural learning, context, head direction cells, hippocampus, place cells, spatial cognition

## Abstract

I have been incredibly fortunate to have worked in the field of hippocampal spatial coding during three of its most exciting decades, the 1990s, 2000s, and 2010s. During this time I had a ringside view of some of the foundational discoveries that were made which have transformed our understanding of the hippocampal system and its role in cognition (especially spatial cognition) and memory. These discoveries inspired me in my own lab over the years to pursue three broad lines of enquiry—3D spatial encoding, context and the sense of direction—which are outlined here. If some of my personal recollections are a little inaccurate (such is the nature of episodic memory!) I apologize in advance.

## 1 Introduction

I vividly remember the first grid cell I ever saw. It was 1994: I was a postdoc in John O'Keefe's lab at University College London (UCL) and I was having a frustrating time trying to get anything sensible out of my entorhinal cortex (EC) recordings. EC is very hard to reach with tetrodes, and I was seeing a lot of theta‐modulated cells, but not much else I could get a handle on. There were also some odd things: a few rhythmic cells that seemed to fire on every alternate theta cycle, and cells that looked like place cells except with multiple firing fields, which were almost circular, and evenly spaced. I collected these in my experimental folder, thought “how odd” and moved on, publishing a small paper on medial septal modulation of entorhinal theta firing (Jeffery, Donnett, and O'Keefe 1995). In one fell swoop I had managed to miss both theta skipping^1^ and grid cells! When recounting this story to students these days I introduce them to Isaac Asimov's famous statement: “The most exciting phrase to hear in science, the one that heralds new discoveries, is not ‘Eureka’ but ‘That's funny…’.”

**FIGURE 1:**
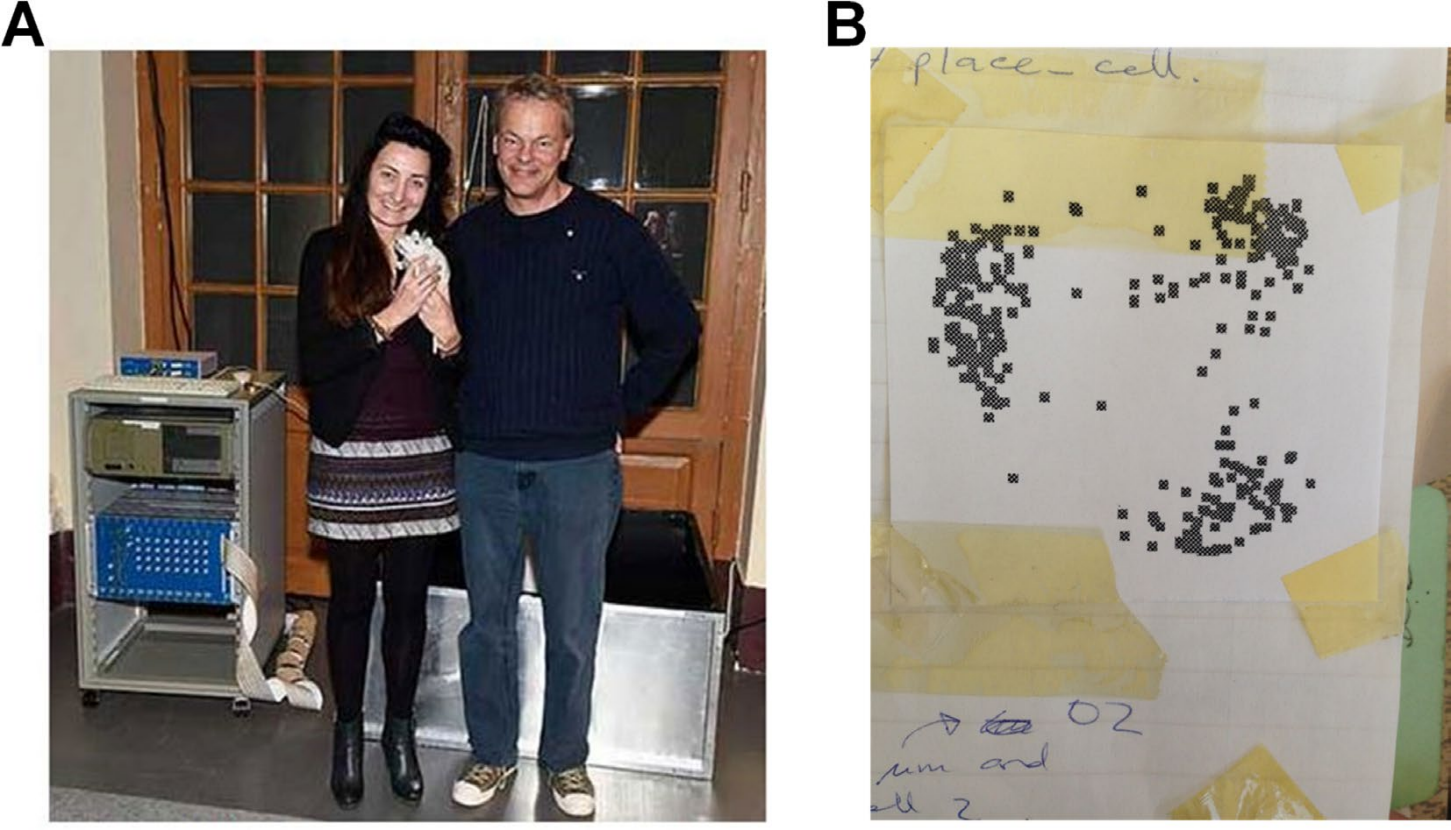
(A) May‐Britt and Edvard I. Moser presenting the recording system on which grid cells were first recorded to the Nobel Museum during the 2014 Nobel Laureates' get‐together on December 6, 2014. Copyright Nobel Media AB 2014. Photo: Niklas Elmehed. (B) I am pretty sure this is a grid cell.

My husband Jim Donnett, meanwhile, also an O'Keefe postdoc, was working on the technical side of things. He and John's electronics technician, Clive Parker, had decided that the existing recording setup, run from a car battery and requiring a spaghetti of cables to route signals from multiple Neurolog amplifiers to their various targets, could be improved by using the then‐newly‐emerging digital signal processing technology that enabled everything to be contained in one compact, cable‐free box. Sometime around then the Mosers, Edvard and May‐Britt, spent time with us and liked the look of the new recording system, so they took one back to their new lab in Trondheim. Ten years later their PhD students Marianne Fyhn and Torkel Hafting properly discovered grid cells with it, and neuroscience history was made. That system now resides in the Nobel museum in Stockholm (Figure 1A). My grid cell recording meanwhile is a Sellotaped piece of paper (Figure 1B) in an old notebook that I like to take out every now and then and ponder wistfully.^2^

In my defense, grid cells are hard to spot when recording environments are small, and I think quite a few of us, exploring EC around that time, had seen multi‐field place activity (see for example Figure 3 in Sharp 1999) but had not recognized it as part of a larger pattern. After leaving John's lab I set up my own lab in the Division of Psychology & Language Sciences, also at UCL, and I recall a journal club at which we discussed an interesting paper of Marianne's, published in 2004, the year before *the* grid cell paper, in which she showed multi‐field cells in medial EC (Marianne Fyhn et al. 2004). I have a clear memory of Tom Hartley, from Neil Burgess's lab, speculating that the distribution seemed a little non‐random and wondering if a regular pattern might emerge if recordings were made in a much larger environment. Legend has it that Bill Skaggs made a similar observation when he saw the data on a poster at that year's Society for Neuroscience meeting, and this is what prompted the large‐environment recordings. Whatever the reality, the fact is that when the Hafting et al. paper (Hafting et al. 2005) dropped, I think most of us in the field were simultaneously flabbergasted and unsurprised. It was “Oh my God” and “Of course!” at one and the same time.

I recall the moment of seeing the iconic grid cell figure on my screen with clarity, and I exclaimed at the beautiful pattern. Caswell Barry, working with both Neil and me, had emailed the pdf around our labs with the understated suggestion that perhaps we should discuss it at the next journal club. I emailed back saying “No way—let's discuss it now!” so we decamped to the Queen's Larder pub with the rest of our teams and spent a few hours poring over the paper and trying to understand what it meant.

We were quite puzzled. It was clear that what we were seeing had something to do with measuring out space: the even spacing of grid‐cell firing fields could mean nothing else. But why, and how? And there was a conundrum, which Neil, Caswell and Tom, who along with Francesca Cacucci had been thinking a lot about place cell metrics (Hartley et al. 2000), immediately spotted. John and Neil had found that in a stretchable box, place fields were somewhat elastic, inasmuch as stretching the box would cause the fields to stretch (O'Keefe and Burgess 1996), although not by as much. Grid cells, on the other hand, seemed to produce a rigid‐seeming, almost crystalline pattern. And yet, it seemed hard to believe that grid cells were not providing the metric inputs to the place cell map (Jeffery and Burgess 2006). So how could these things be reconciled? Caswell and another student, Robin Hayman, went back to the lab and began recording grid cells in the same stretchy box, to see whether grid‐cell grids were really as rigid as they seemed. In another amazing display of perspicacity I advised against this experiment, being sure that they would never be able to see such a subtle effect in such small environments, and also being skeptical that such an effect even existed. I was wrong of course, and they published a beautiful paper a couple of years later showing that grids stretch in just the same way that place cells do, so long as the environment is familiar (Barry et al. 2007). The dependence on familiarity, as well as the incompleteness of the stretching, points to an interaction between the (learned) anchoring effect of environmental cues and the (intrinsic) updating effect of self‐motion (path integration) cues (Redish 1999, Chapter 9) that is evidently a fundamental property of allocentric spatial processing in the brain.

### 1.1 Grid Cells in Vertical Space

The firing patterns of grid cells are very striking and pretty but they also offer an experimental opportunity, because they provide a window into the metric foundation of the cognitive map. In my lab we decided to use this tool to explore whether the cognitive map permeates, as it were, three‐dimensional space. In other words, does a grid cell produce its pattern throughout a volume, if given the opportunity, or does it merely plaster it over the environment surface like a polka‐dot blanket? Or, less likely but still possible, might it even track only pure horizontal distance irrespective of the environmental undulations?

**FIGURE 2:**
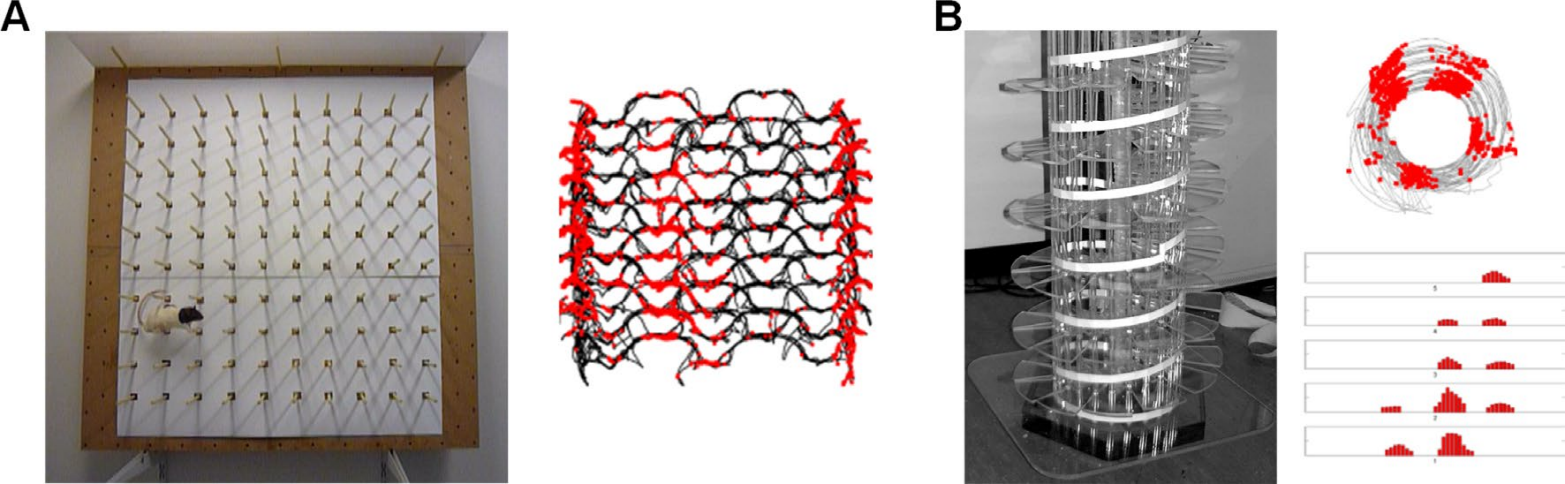
My lab's first attempts at recording in 3D mazes. (A) The pegboard. The photo shows a rat from one of our behavioral experiments foraging over the area while standing on the pegs. The plot on the right is a spike plot from an entorhinal grid cell showing the path of the exploring rat over the course of a trial as a black line, and the spikes from the cell as red dots. Note that in the horizontal dimension the firing is interrupted, whereas in the vertical dimension it is continuous, resulting in vertical stripes. (B) The helical maze. The photo shows the assembled maze. On the right is a spike plot from a grid cell, shown from two viewpoints: overhead (top) and side‐on with the coils unwound and firing now expressed as a rate histogram (bottom). As with the pegboard, the firing seems interrupted when considered in the horizontal plane, but mostly uninterrupted in the vertical dimension (the apparent interruption is due to positional changes as the rat hugs the central pillars increasingly closely as it gets higher).

These questions are not easy to address in rats, which don't move very freely through a volumetric space,^3^ but as it happens we had already begun to explore 3D processing in rat place cells. This experiment had started essentially because we had a hammer and needed a nail to try it out on. Jim had teamed up with André Fenton and others to form a company called BioSignal that developed (among other things) a digital telemeter for single neuron recording (Fenton, Jeffery, and Donnett 2011). We had been pondering what kinds of experiments one might do without the constraint of recording cables. One obvious answer is recording in complex 3D space, and both André and I had been thinking independently about trying this. In my lab we had started training rats to explore a climbing wall studded with projecting pegs (the “pegboard”, designed and built by an undergraduate student, Emma Leeper; Figure 2A), while in André's lab, Edo Keleman had designed an architecturally wondrous piece of apparatus which we called, accurately but unimaginatively, the “helical maze” (Figure 2B)—a Plexiglas spiral staircase made from dozens of interlocking steps that wound in coils from the ground up to about 2 m high. André had decided he didn't have the capacity to do experiments with this apparatus so he brought it in pieces over to the UK in his airplane luggage and we set it up in my lab. Madeleine Verriotis began recording place cells on it (although not, as it happens, with the telemeter). Meanwhile, in the room next door Aleks Jovalekic and Robin Hayman had started recording cells from rats foraging on the pegboard, which they did relatively freely. With both of these structures the emerging picture with place cells seemed to be that they produced firing fields in the vertical dimension that were similar to those in the horizontal, if slightly elongated (Hayman et al. 2011). Then grid cells came along and we decided to see what they would do. Would they track vertical travel distance?

The first pegboard grid cell image dropped into my inbox late one evening while I was checking email in bed (a bad habit I still have) and I was very surprised to see not circular grid fields, and not disorganized or absent firing, but rather *stripes*, running vertically from the bottom to the top of the arena (Figure 2A). Shortly afterward Madeleine found the same thing on the helical maze (Figure 2B)—grid cells expressed their usual circular and spaced‐apart firing fields when the pattern was viewed from above, but when viewed from the side then the cells could be seen to fire on every coil of the helix. In both cases, it seemed that the cells were not tracking the vertical distances moved, but only the horizontal ones. We were forced to consider the surprising possibility that the cognitive map might contain no height information and simply be a mosaic of flat maps (Jeffery et al. 2013). Following later experiments I have come to moderate this view, but only partially.

The finding also revealed a disconnect between what grid cells were doing and what place cells were doing, which contradicted the prevailing assumption that grid cells drive place cells. In fact, much evidence now supports that the interaction between place and grid cells is complex and bi‐directional (Bush, Barry, and Burgess 2014; Morris and Derdikman 2023)—another pervasive property of spatial networks (and many other cognitive systems).

There are several possible reasons for the failure of our grid cells to track vertical movement, and it took us a while to untangle these. The pegboard stripes are reminiscent of earlier observations by Dori Derdikman, then in the Moser lab, that grid cells would produce stripes running across the “grain” of a zig‐zagging hairpin maze (Derdikman et al. 2009). Perhaps the pegboard stripes are a similar phenomenon (Ulanovsky 2011). However, in the hairpin maze rats could move only in one direction at a time, being constrained by boundaries, whereas the pegboard rats could move in any direction over the surface, although they did move differently in the two dimensions. It seemed that these could be different issues, albeit both related to the dimensionality of travel.

**FIGURE 3:**
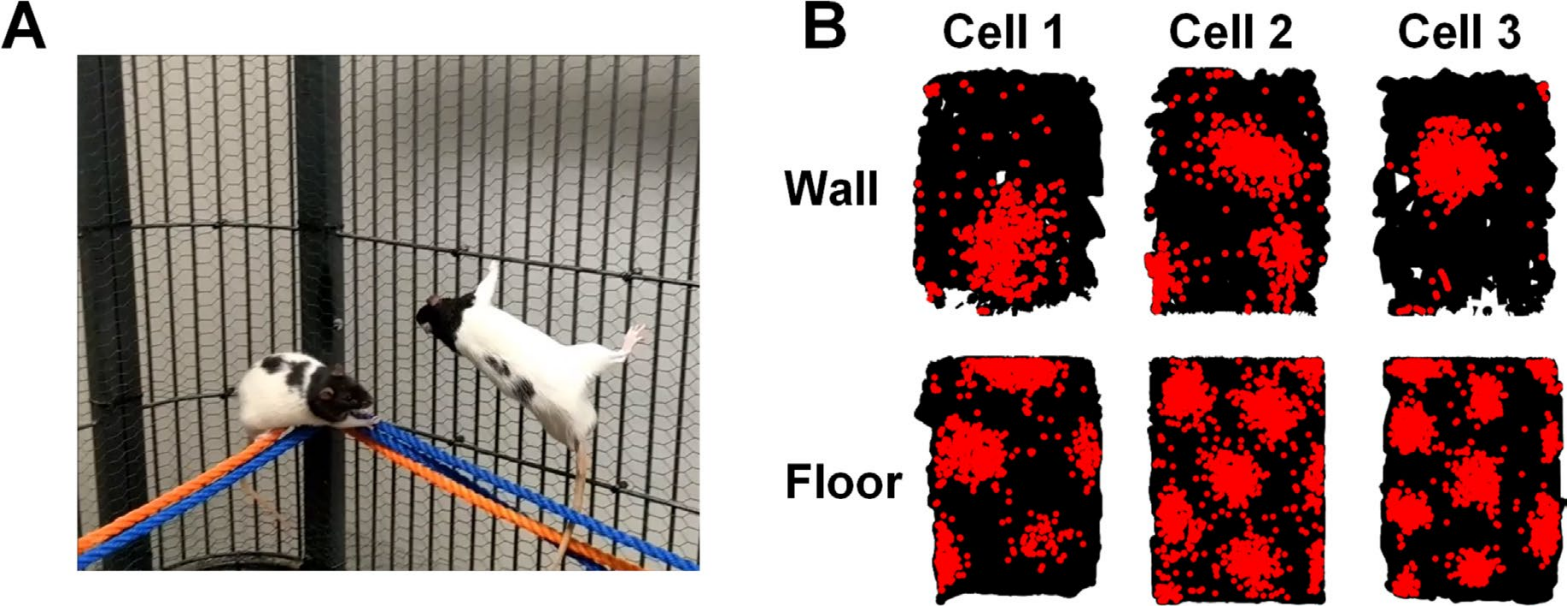
Grid cells in climbing rats. (A) The rats were raised in a large cage providing multiple climbing opportunities to ensure full 3D competence. (B) Spike plots (as in Figure 2) from three grid cells recorded on either a floor or a wall. On the floor the cells produced their characteristic regular array of firing fields (“blobs”), but on the wall they typically produced only one, or a few, fields with little evidence of a regular pattern.

To better isolate the reasons for the vertical stripes we decided to alter the relationship of the foraging animals' bodies to gravity. Giuilo Casali led on this project. First, to rule out dimensional inexperience as a possible reason for the absent vertical periodicity he gave the rats a huge parrot cage to live in, filled with climbing apparatus (Figure 3A). The animals love living in this environment and become very experienced climbers and jumpers (which makes recording with them quite challenging). We then considered whether gravity might account for stripes vs. blobs in the different planes. Robin had found that normal grids would be produced on a slanted plane just as if it were flat, suggesting that the grids aligned with the arena surface and not with Earth‐horizontal (Hayman et al. 2015). Giulio added chicken‐wire to his recording arena so that the rats could roam freely over the surface even if it were tilted all the way to the vertical. He found that grid cells now produced blobs (Figure 3B) instead of stripes (Casali, Bush, and Jeffery 2019). So, it wasn't gravity (or rather the relationship of the rat's body to gravity) that accounted for the pegboard stripes. Interestingly, however, the blobs were expanded and did not appear to be regularly arranged, although it was difficult to tell. Giulio and Dan Bush, in Neil's lab, undertook a detailed analysis that suggested that the expansion might be due to a blunted speed signal on the wall, as if the cells treated the rat as moving more slowly than it really was, thus firing over a larger distance.

Whatever the reason for the expanded pattern, these observations ruled out the relationship of the body to gravity as the determinant of blobs versus stripes on a vertical surface. Together with Derdikman's findings, they suggested instead that it is the freedom of movement in a given dimension that allows grid cells to track distance in that dimension. If this is the case, then if rats can move freely in all three dimensions, grid cells should now produce discrete firing fields in three dimensions—spheres. Furthermore, theoretical considerations suggest that these should be arranged in a regular pattern called a hexagonal close‐packed array (Jeffery et al. 2013, 2015; Stella and Treves 2015). To test this prediction, Roddy Grieves set to work recording wirelessly as rats climbed through a 1.2‐m‐cubed lattice made from rods. Aleks had previously shown that rats could navigate to a location within this lattice (Jovalekic et al. 2011) and so we already had some sense that rats knew where they were within this space. But what would place and grid cells do? It took 5 years to find out, and the result was slightly surprising.

Roddy, aided by Sophie Renaudineau and a team of students, began with place cells, and found that they indeed would form globular fields that were distributed evenly throughout the space (Grieves et al. 2020), so the cells could evidently self‐localize. Michael Yartsev in Nachum Ulanovsky's lab had reported a similar finding for flying bats (Yartsev and Ulanovsky 2013) so we were happy about this consistency. Roddy then turned to grid cells and found that, like place cells, the cells formed multiple, globular and evenly distributed firing fields (Grieves et al. 2021), again pointing to a self‐localization capability by the cells. Surprisingly, however, he found that although the distribution was uniform it was not regular. Thus, the grid cells had preserved their self‐localization capability, and their propensity to fire in discrete blobs, but had abandoned any semblance of symmetry. This led us to a heretical thought—perhaps the symmetry of grid cell firing is not necessary for their function? Might it be an epiphenomenon?

Lending support to this epiphenomenon notion was the simultaneous report by Gily Ginosar, in Ulanovsky's group, that medial entorhinal neurons in bats also formed discrete irregularly arranged blobs as the animals flew about in a volumetric space (Ginosar et al. 2021). Gily found, however, that unlike in our rats, the pattern was not completely random: there was a local order, inasmuch as the distances between pairs of firing fields were more similar than would be expected by chance. This finding of varying degrees of orderliness is reminiscent of the spontaneous behavior of other self‐organizing natural systems. For example, silicon dioxide organizes in some conditions into a regular crystal, called quartz, or in other conditions into a completely random organization, called amorphous silica. There is also an intermediate state, glass, which occurs under some crystallization conditions and results in local order between tetrahedral structures in the network, but no overall long‐range regularity. We speculated, then, that something similar happens with grid cells: whether they produce a symmetric or irregular pattern, or something in between, is simply a function of their formation conditions—the relevant conditions in this system being the freedom of movement afforded by the different dimensions of the environment.

If grid cells do not obligatorily produce symmetric firing patterns, then how can they be used for spatial computations? A spatial system that only worked in a flat open field would be fairly useless in real life, so we assume that the irregular grids in volumetric spaces are also useful, given that rats and bats can navigate perfectly well in these spaces. One possibility is that perhaps a path integrator can do quite well with a grid cell input even if the fields are irregularly dispersed, provided that the *overall* statistics of their size and spacing are consistent. Given such consistency, it should still be possible for the system to work out roughly what distance was traveled. Analogously, an optic flow field works just as well for motion computation if the pattern is irregular as if it is regular. One possible reason for the firing field discreteness of grid cells might therefore simply to create sheets of “speckles” at varying scales that can be used for, as it were, the spatial version of optic flow.

The end conclusion of all of these experiments is that… it's complicated. Grid cells clearly are predisposed to fire in an interrupted fashion along axes of free travel, and sometimes in a continuous manner (as if not tracking distance traveled) along an interrupted axis. But the vertical travel was, arguably, equally interrupted in the pegboard and lattice maze environments, and yet firing fields formed blobs (not stripes) in the latter. This might be because the lattice has more degrees of movement freedom and thus less vertical self‐similarity, in the unfolding experience of the initially exploring rat. Whatever the exact reason, it is clear that grid cell firing patterns are heavily influenced by the environment's movement affordances. What this says about their function, though, is anybody's guess.

### 1.2 Context

**FIGURE 4:**
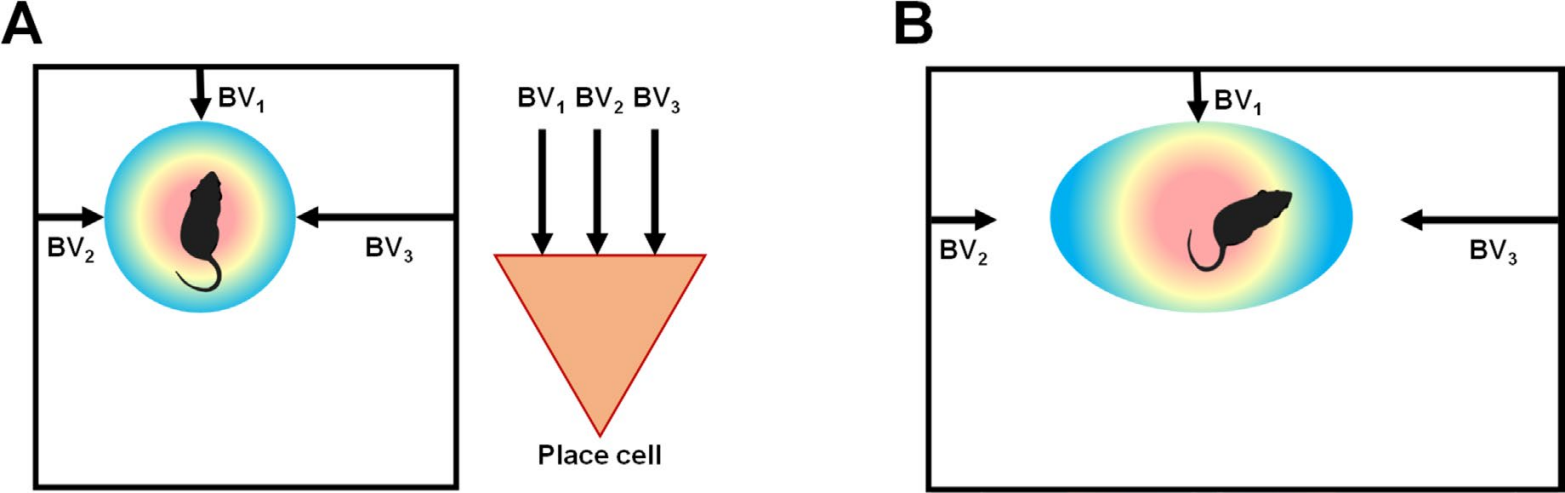
The boundary vector model of Burgess and colleagues. (A) A place cell receives multiple inputs conveying information about the distance and direction of the nearby boundaries. At the point where the inputs converge, a place field (colored disk) forms. (B) If a familiar environment is stretched, pulling opposing boundary vectors apart, the field also stretches in that dimension. Note, however, that the stretching is incomplete—something else (self‐motion information) opposes the stretching.

Grid cells fire in every environment but place cells in only some, so the brain evidently has a mechanism for determining which environment—which *context*—it is in. I first became interested in the contextual influences on place cells while in John's lab. At around this time his stretchy‐box experiment with Neil was unfolding, and it was becoming apparent that place cells are sensitive to the boundaries of the environment (Barry et al. 2006; O'Keefe and Burgess 1996). Neil and John proposed that a place cell adds together a number of “boundary vectors” (Figure 4): hypothetical inputs that signal both distance and direction of nearby boundaries. When the box is stretched, a given cell's boundary vector inputs no longer intersect at the original place field location and so the field gets pulled apart.

Meanwhile, work from Bob Muller's lab (see review in Kubie 2025) had shown that place cells change their firing patterns—“remap”—if the environment is changed (Muller and Kubie 1987) and furthermore that this change does not have to be in the size/shape of the environment but can just be in its appearance (Bostock, Muller, and Kubie 1991). Putting these two observations together, it seemed to me that insofar as place cells are sensitive to boundaries, they must sensitive to the color of the boundaries—the postulated boundary vectors must be color‐coded.

When I set up my own lab at UCL, my first experiment aimed to test this hypothesis. My reasoning was that if boundary vectors are color‐coded, then by combining boundaries from two differently‐colored familiar environments, we should be able to trick a place cell into producing its field in a new location. For example, if a cell's place field was at X_1_Y_1_ in context 1 and X_2_Y_2_ in context 2, we should be able to induce it to fire in X_1_Y_2_ or X_2_Y_1_, with appropriate re‐combining of the boundaries. Mike Anderson started trying this out—and it failed completely. Although the cells remapped robustly to changes in the color of the box, they did not in fact care at all what we did with the walls. The only thing they seemed to respond to was the color of the *floor*. There was thus a disconnect between the influence of the boundaries, which position the fields, and the color of the floor, which determines *which* fields get positioned (Jeffery and Anderson 2003). It seems that the boundary vector inputs are not color‐coded after all.

**FIGURE 5:**
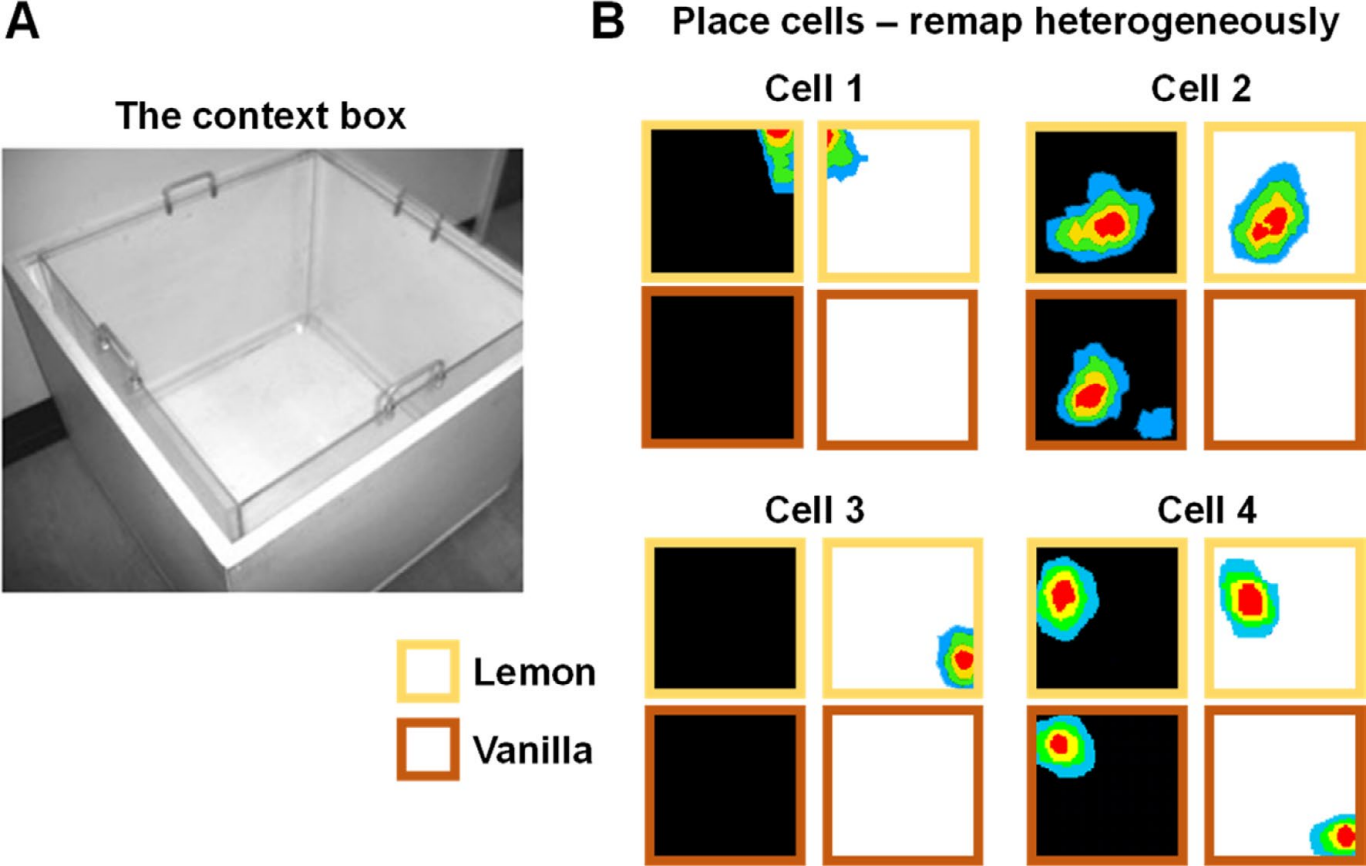
Remapping of place cells in response to context changes. (A) The “context box” has an outer casing painted white (as in photo) or black, and an inner Plexiglas insert scented with lemon or vanilla food flavoring. (B) Four simultaneously recorded place cells tested in the four different odor‐color combinations in the experiment by Anderson et al. (2006), producing firing fields shown here as heat plots (red = max. firing). Note the heterogeneity, or “partial remapping.”

Mike then started playing with the contextual cues further, adding a second dimension in the form of odor. The original reason for this experiment, which became lost in the mists of time, was to test my suspicion that much “visual” remapping we were seeing in the lab was actually due to olfaction: black and white paint simply smelling different. To manipulate these dimensions independently Mike added to the apparatus a Plexiglas inner chamber that could be scented with lemon or vanilla food flavoring (Figure 5A), which sat inside a wooden black‐ or white‐painted outer casing. The “context box” could thus be black‐lemon, black‐vanilla, white‐lemon or white‐vanilla.

As usual, I was very quickly disavowed of my original hypothesis, as place cells remapped considerably *more* robustly for vision than for odor. However something odd was happening that we hadn't expected, which is that not all the cells remapped to any given change. Only some did: a different subset for the different changes (Figure 5B). This was surprising, because the prevailing attractor theory of place‐cell maps (Samsonovich and McNaughton 1997; see review in McNaughton 2025) predicted that if some cells remap then the whole system should—there should be global remapping. This clear demonstration of partial remapping thus cast doubts on the attractor model. It seems that the system has a propensity to globally remap (or “pattern complete”) but that mixed states can occur reasonably easily, and may have the function of representing mixed contexts (see also Redish 1999; Chapter 9; Redish and Touretzky 1997; Touretzky and Redish 1996; Jeffery 2011). In support of this, Mike showed that rats can express mixed behaviors in a given setting, indicating that they know both that an environment is familiar but also that it has changed (Michael I Anderson et al. 2006). This built upon our earlier finding that rats can express a well‐learned navigation task even when the entire pattern remaps (Jeffery et al. 2003), and also that the goal location does not appear to influence the place cell map (see Duvelle et al. 2019). Eleonore Duvelle also showed that the map also does not appear to encode environmental connectivity (Duvelle et al. 2021). These and other findings have led me to the unpopular view that locational value is not directly encoded in the place cell map.

**FIGURE 6:**
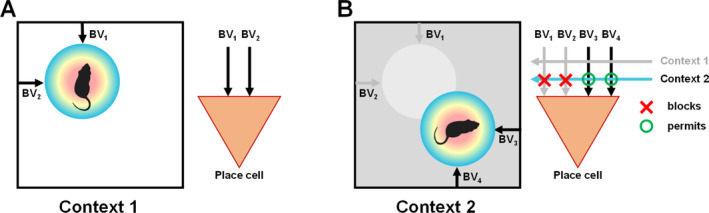
The boundary vector model of Burgess and colleagues, and our contextual gating adaptation of it. (A) The original model (see also Figure 4) proposed that a place cell receives boundary vector (BV) inputs that combine to specify a precise location of the firing field (colored disk) relative to the environment boundaries. (B) The contextual gating model addresses how the BVs could function in multiple contexts. It proposes that a cell has multiple sets of BV inputs, each specifying a field location, and these are gated (blocked or permitted) by convergent contextual inputs. Depending on which contextual inputs are active, the appropriate set of BVs will drive the cell and position its field appropriately for that context.

Partial remapping was not the only oddity in Mike's data. He found that although some cells would remap only to one or other of the context dimensions (mostly to color as mentioned above), the vast majority were sensitive to some unique combination of color and odor (Figure 5B; M I Anderson and Jeffery 2003). These cells thus showed “conditional remapping,” only remapping in response to a change in a given context cue if another context was also present. Robin similarly was finding that a place cell would remap to a change in color only if the box was in one location in the room and not if it was in a different one (Hayman et al. 2003). This context dependence did not, interestingly, pertain to directional cue learning, as shown by Subhojit Chakraborty (Chakraborty et al. 2004). Also, we never saw (then or since) any case where a cell with a field in one context and a different field in a different context would sometimes express both fields. It is almost as if there is an either‐or switch between sets of place cell inputs (Figure 6).

Based on these observations we proposed a modified version of the boundary vector model, called the “contextual gating” model, which attempts to explain such switching behavior (Jeffery et al. 2004). It supposes that context‐free boundary vector inputs impinge on a place cell, but these can only drive it to its firing threshold *if* the correct context cues are also present—these act as a gate.

Later on, after grid cells were discovered, we further developed this model to propose that the boundary vector inputs are routed through the MEC grid cells to the dentate gyrus, and the context cues arrive via the lateral EC (LEC) inputs (Hayman and Jeffery 2008), terminating closer to the soma, so that they can control (gate) whether the spatial grid‐cell drive can get through to the soma. The LEC inputs thus determine which subsets of grid cells drive a place cell in a given context. This model proved effective at simulating place cell conditional remapping, and also in reproducing some intriguing dentate gyrus results of the Leutgebs' (Leutgeb et al. 2007). With the new dendritic voltage imaging methods that are steadily being refined, it is hoped that this model can 1 day be tested with real data.

### 1.3 Context and Grid Cells

**FIGURE 7:**
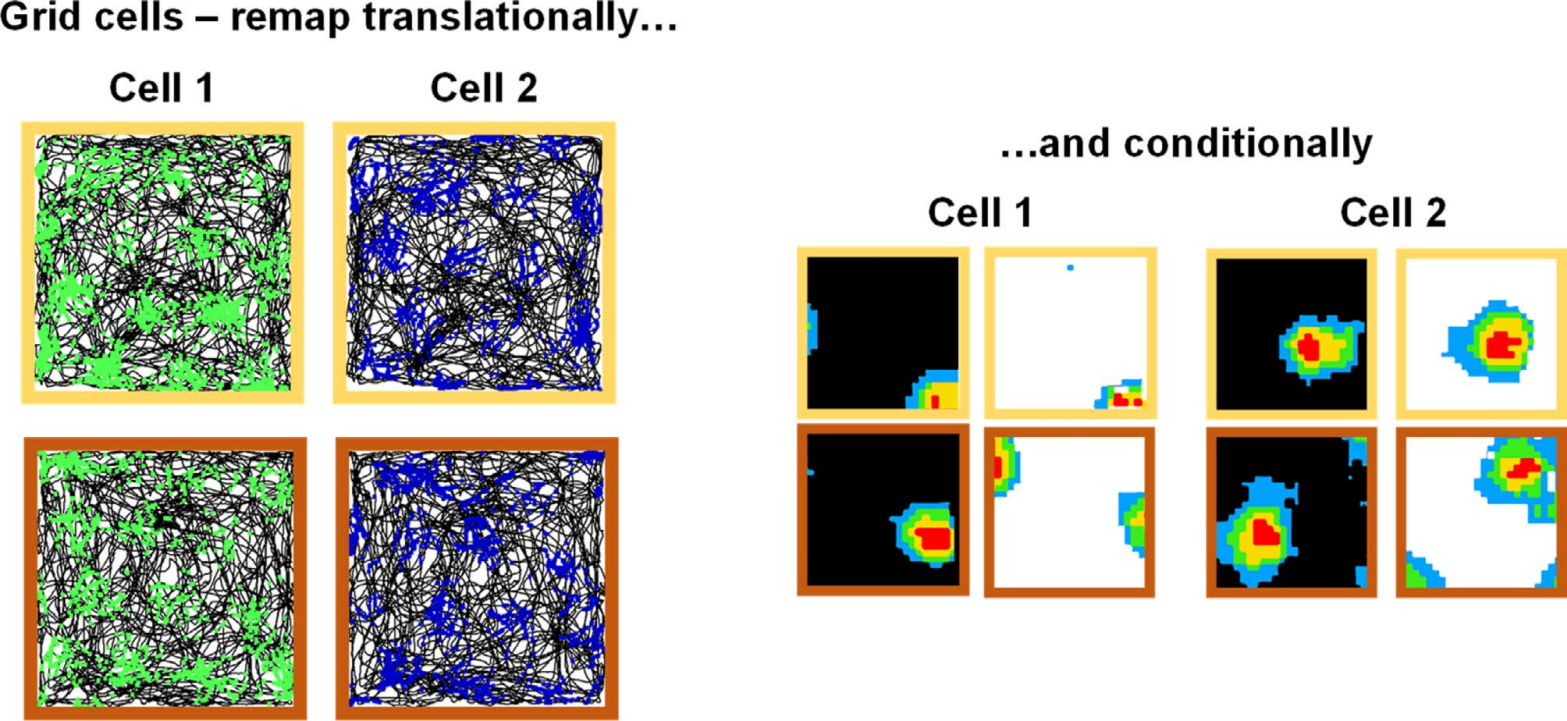
Contextual remapping in grid cells. (Left) Spike plots of two grid cells simultaneously recorded in the context box in the experiment by Marozzi et al. (2015). Note the very subtle shift of the grid, but no rotation, in response to the odor change. (Right) Heat plots of grid cell firing in the context boxes, painted black or white as shown in the figure, and scented lemon (yellow border) or vanilla (orange border). These cells, like the place cells in Figure 5, showed conditional remapping—the remapping induced by changing one color depended on the odor present, and vice versa.

When grid cells came along, we wondered whether these cells would also be sensitive to changes in context. Early results from Marianne Fyhn seemed to suggest that this is so (M Fyhn et al. 2007), with grid cells both translating and rotating their grids when the context changed. In my lab, Liz Marozzi, Lin Lin Ginzberg and Andrea Alenda therefore investigated whether we would see the global responding to color‐odor context configurations that we had expected (but not found) in our place cells and whether, if so, this would be conditional. Slightly to our surprise, we found that grid cells did remap globally (all cells together), but only in the subtlest imaginable way—they translated their fields slightly (and, interestingly, conditionally; Figure 7), but did not rotate them (Marozzi et al. 2015). This raises the question of why the place cells don't translate *their* fields with these changes. The simplest answer is provided by the context gating model: the change in context alters (via gating) which grid cells drive a given place cell.

What causes the grids to translate? One possibility is that it is the remapped place cells! Although this seems a little chicken‐and‐egg (G. Morris and Derdikman 2023), we know there are plentiful feedback inputs from the place cells to the deep layers of entorhinal cortex which potentially account for many grid cell findings (Bush et al. 2015). In support of this possibility, Derdikman's group analyzed our data further and showed that although the spatial remapping of the grid cells is minimal, there is widespread rate remapping across the firing field array, inasmuch as the variable intensity of individual grid fields, which is stable within a context, remaps across contexts (Ismakov et al. 2017). This point‐like reorganization suggests that the altered place cell pattern (a place cell being a “point”) projects slightly differently onto the grid network, redistributing the heterogeneous intensity (and hence field rate) and also dragging the grid pattern sideways slightly.

A further implication is that the head direction signal, which doesn't change under contextual remapping, orients the grids independent of the place cell inputs. This then brings us to the head direction cells.

### 1.4 Direction

The boundary vector model of place cell positioning assumes the existence of a directional signal, which provides the directional part of the boundary vector, and this information likely comes via the head direction (HD) cell system, which was first reported by Ranck, Taube and colleagues (Ranck‐JB 1984; Taube, Muller, and Ranck 1990a, 1990b). I became interested in the role of the directional signal when I was in John's lab, recording place cells in a cue‐controlled environment and finding that just rotating the internal direction sense of a rat is enough to rotate its place fields (Jeffery et al. 1997). I also found that the cells could learn about both the appearance and reliability of the visual cues that contribute to place field orientation (Jeffery and O'Keefe 1999; see also Knight et al. 2013; Page et al. 2014) as did Jim Knierim, then in Bruce McNaughton's lab (Knierim, Kudrimoti, and McNaughton 1995). Later on, when my lab at UCL started to explore HD cells further, Yave Lozano showed that they can discriminate visual patterns to quite a high degree of resolution (Lozano et al. 2017) via a pathway that James Street later found depends on the thalamic dorsal lateral geniculate nucleus (Street and Jeffery 2024). We had also earlier found, contrary to expectation, that they aren't strongly influenced by environment geometry. This was shown by Rebecca Knight and Caitlin Piette who found that the cells would best orient according to geometry if the rats are mildly disoriented first (Rebecca Knight et al. 2011). When we started becoming interested in 3D space, Jonathan Wilson and Hector Page, showed that HD cells can compensate for movements in 3D space so as to maintain a horizontally‐referenced directional signal (Page, Wilson, and Jeffery 2018). HD cells are therefore an excellent model system with which to investigate sensory integration.

Somewhere around my time in John's lab I had also stumbled upon another brain structure, retrosplenial cortex, quite by accident. Jim and I had been playing around with retrograde tracing, using Fast Blue in an attempt to try and find the source of the elusive “speed cells” that John and Jim had once spent a happy afternoon recording a single example of (see Figure 5 in O'Keefe et al. 1998).^4^ On one of the slides we saw a huge amount of fluorescence in the neocortex. I don't remember where the injection site was but I do remember saying “What the heck is that?” and looking it up in Paxinos and Watson—it was something called retrosplenial cortex (RSC), which I hadn't heard of. I filed it away in my brain, moved on, and didn't come back to RSC for about 20 years.

Shortly thereafter, McNaughton's group reported the finding of head direction cells in RSC (Chen et al. 1994), and a few years later Pat Sharp's group published a more detailed study of them (Cho and Sharp 2001; see review in Sharp 2025), and so later on, when I started exploring the pathway for visual information coming into the hippocampal network, I became more interested in RSC. I recruited a postdoc, Pierre‐Yves Jacob, to record HD cells in RSC. To get things started, I suggested that he replicate, in these RSC HD cells, an experiment that Hugo Spiers had earlier done with place cells. Hugo's experiment had investigated whether place cells could use non‐visual information to distinguish visually identical but spatially separated environments (Spiers et al. 2015). In that experiment, to our surprise, place cells had not made this discrimination despite abundant self‐motion information that could have helped them. Francis Carpenter in Caswell Barry's then‐newly‐established lab also showed this for naïve grid cells (but not familiarized ones; Carpenter et al. 2015). This suggested that environmental cues are very dominant in their influence.

**FIGURE 8:**
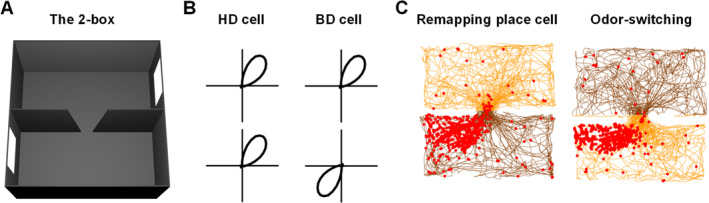
Spatial cells in the 2‐box. (A) The 2‐box is made from two rectangular compartments connected by a central doorway, each possessing a cue‐card at one end. The boxes are visually identical so the visual scene alone cannot be used to determine which direction is which, of the two possibilities. However, the compartments are scented with lemon and vanilla so that the brain in principle has the information needed to distinguish them. (B) HD cells can use this information and produce tuning curves with the same orientation in both compartments. Bidirectional (BD) cells, by contrast, rotate their tuning curves 180° between the compartments, suggesting that they use environment layout but not odor to orient. (C) Spike plots of a place cell from the experiment by Cheng et al. shown as previously except that the paths are colored yellow and brown for lemon and vanilla compartments, respectively. Place cells remap between compartments (in this example, firing only in one compartment), suggesting sensitivity to the odor context. However, sometimes they invert the contexts, in a process we call “odor‐switching.”

To repeat this experiment with HD cells we couldn't simply spatially separate the compartments, because HD cells in a new environment spontaneously adopt the same firing direction throughout a connected multi‐compartment space (Taube and Burton 1995). We could, however, *rotate* the compartments, which would introduce an analogous conflict between the visual scene and the internal direction sense. Pierre‐Yves thus created two visually identical compartments that were directionally distinct, one 180° rotated with respect to the other. He also used the non‐visual cue, our traditional vanilla and lemon odor contexts, to distinguish the compartments. In this apparatus, which we call the 2‐box (Figure 8A), Pierre‐Yves found that HD cells could tell the environments apart reasonably well (Jacob et al. 2017). Thus, a north‐signaling HD cell (for example) would fire when the rat faced toward a northern cue card in the lemon compartment and away from an identical southern cue card in the vanilla one, even if the rat were just randomly placed in one or other compartment. This didn't happen all the time (a point we come back to later) but it was well above chance.

So HD cells can respond to odor and use it to interpret a visually ambiguous space: so far so good. But there was another, more surprising finding lurking in the data: quite a few of the cells didn't make this disambiguation (Figure 8B), flipping from one direction to the other when the rat stepped through the doorway. Furthermore, these were all only in the dysgranular (more superficial) region of RSC.

These were clearly a new cell type, which we called, for want of a better word, “bidirectional” (BD) cells. Sometime later Ningyu Zhang found cells with four tuning curve directions in an environment made from four boxes (Zhang, Grieves, and Jeffery 2022), so now we call them “multidirectional” (MD) cells. We also found evidence that these cells learn an association with HD cells after the rat has had experience in the environment. Hector modeled this scenario and showed that a learned interaction between visually responsive cells and HD inputs can in principle generate both types of cell (Page and Jeffery 2018). The overall conclusion is that this region of RSC is plastic, and mediates between the learned visual scene and the main HD signal via these visually controlled HD‐like cells. Since the viewpoint scene is an egocentric (viewpoint‐dependent) signal and the HD system is an allocentric one, these cells also mediate between egocentric and allocentric spatial processing: something that Neil and colleagues had long ago proposed as a core function of RSC (Burgess 2008; Byrne, Becker, and Burgess 2007).

### 1.5 RSC And Place Cells

Does this ambiguous directional signaling in RSC find its way into the place cells? To find out, Dot Overington and Han Cheng recorded place cells in the 2‐box (Figure 8C). The cells showed, as we had come to expect from these manipulations, partial remapping—they sometimes repeated their fields between the compartments and sometimes remapped. Notably, however, they never *rotated* their fields. Thus, the directional ambiguity had been resolved (we assume by the HD system) before reaching the place cells.

This place cell behavior seemed, on the face of it, consistent with our working model of boundary vector cells that are gated by context and modulated by the direction of boundaries, but there was just one slight niggling issue. This is that every now and then, the whole place field ensemble would disconnect from the odor cues and rotate en bloc. Han called this “odor‐switching.” At first sight this reversal seemed unsurprising, because we already knew from Pierre‐Yves' experiment that the orienting of HD cells, and thus the boundary vectors, by odor sometimes failed. Something didn't quite add up though, and it took me a while to understand what was troubling about this observation.

**FIGURE 9:**
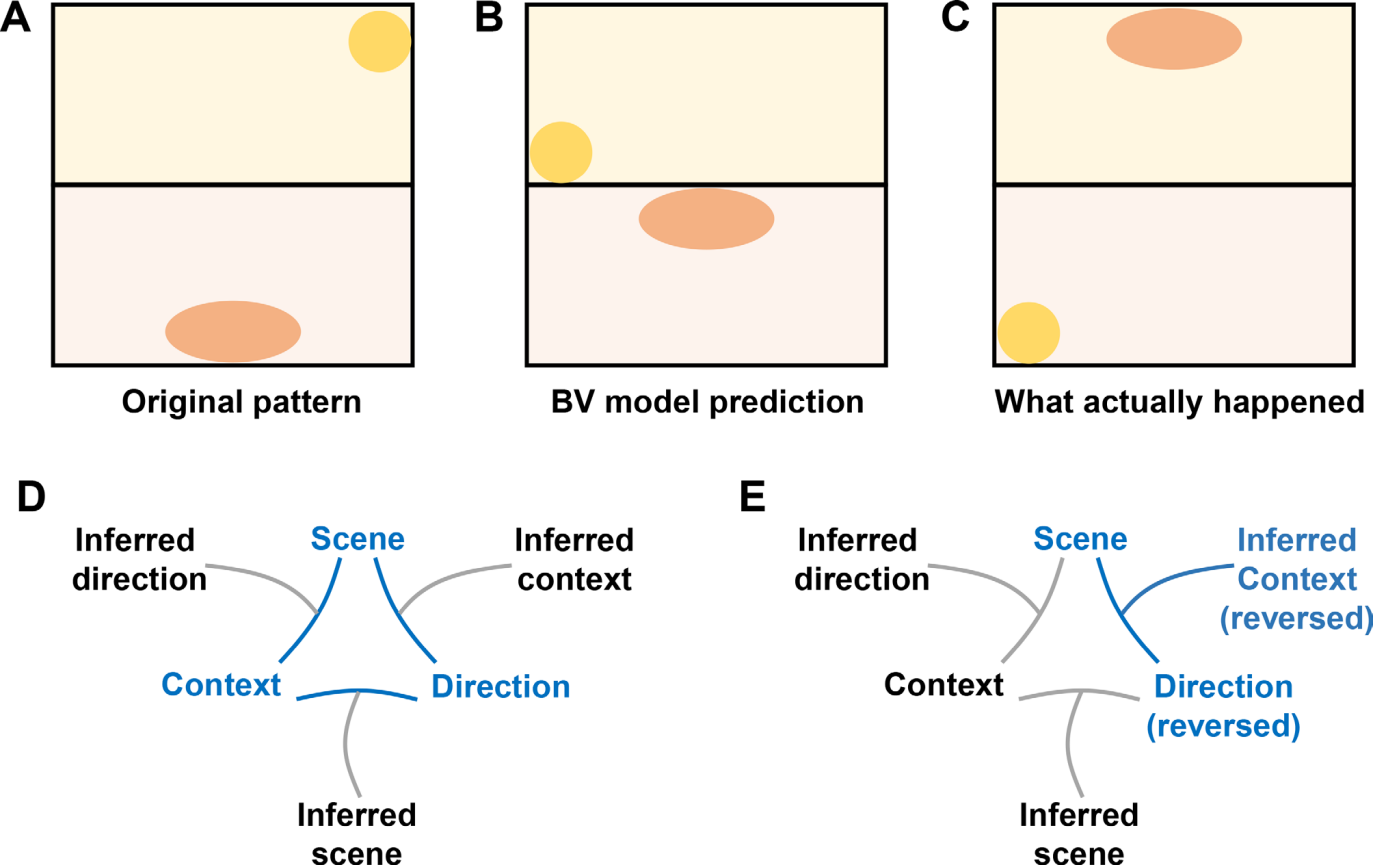
Odor switching and a possible explanation for the pattern rotation. (A) Original place cell pattern in the lemon and (rotated) vanilla boxes. (B) What the BV model predicts if the HD signal disconnects from the odor context and switches directions (local rotation within each context). (C) What actually happens (global rotation across both compartments). (D) A possible model for how the three cue types (context, visual scene and direction) interact—blue denotes active signal and gray denotes inactive. (E) If the context signal drops out and the HD signal disconnects (reverses), the systems infers (reversed) the contexts based on the other two signals, and the whole pattern consequently reverses.

The issue has to do with disconnect between context and boundaries we had discovered many years previously (Jeffery and Anderson 2003). We at first assumed that our HD cells in the 2‐box odor‐switched because the cells retrieved the wrong heading for that context and reversed direction. This would mean that each place cell is getting a “wrong” HD signal. But according to the BV model the fields should therefore just rotate within a given compartment, as in Figure 9B. They don't do this, though—they coherently swap their context patterns (Figure 9C), as if their global context inputs have exchanged identities. But how could that happen? Loss of context signal due to a perceptual failure is one thing, but a *swapping*? How could the brain mistake vanilla for lemon?

We came up with the following explanation, which proposes that the context signals gating the boundary vectors do actually swap identities, due to a mediating abstract context signal, which we might call “inferred context,” which is slightly decoupled from direct perception. The explanation runs as follows: when the rat first enters the environment it has an arbitrarily oriented head direction signal which becomes associated with the visual scene and with the prevailing context cue, to form a three‐way association between these signals (Figure 9D). For example, when the rat is placed in the lemon box facing the cue card, and its north HD cell is active, these elements become associated. Now, having established this three‐way relationship, two of these signals can be used to infer the third. If the rat finds itself facing the cue card and its north HD cell is firing then it must be in the lemon box (even if it couldn't at that moment perceive the odor) and so on. The inferred context is thus a kind of lemon proxy.

We can now explain odor switching as the system having inferred the wrong context: for whatever reason the odors were not properly detected on environment entry and failed to control the head direction signal, which adopted the wrong direction. This then caused inference of the wrong context, which gated the wrong place cell inputs. The reversed HD signal thus reverses the place cell directions, while the wrongly inferred context caused by that reversal retrieves, in turn, the wrong place‐cell pattern. As a result of these two processes, the place fields both rotate direction *and* change sides of the 2‐box, thus rotating en bloc 180°.

The above explanation, postulating an inferred proxy signal, is part of a larger emerging story: the brain can use multiple sources of information to inferentially fill in gaps in its perception. Indeed, it seems increasingly likely that this is *most* of what the brain is doing most of the time: the amount of information that comes in directly through the senses is vastly outweighed by the amount that is routed through inferential systems, like the one described above. Predictive coding is one example of such inference; so is top‐down modulation.

### 1.6 The Thalamus and Inferential Coding

Inferential coding is where my research is currently heading, inspired by the above experiments of inferential cue retrieval, but also by our recent unexpected finding of place cells in ventral anterior thalamus (Lomi, Jeffery, and Mitchell 2023). This discovery was made by Eleonora Lomi, working in my collaborator Anna Mitchell's lab in Oxford, who was recording the thalamic RSC afferents in hopes of getting clues as to why we only see MD cells in dysgranular, and not granular, RSC. Anterior thalamus sends very strong projections to RSC, via two loops (Lomi et al. 2021) so it seemed a good place to look for an answer. The place cells that Eleonora stumbled on were unexpected—but also not completely surprising, as this was not the first observation of thalamic place cells (see Jankowski et al. 2015). Which raises the question: why should thalamus care about location, and why do these reciprocal loops exist?

Our working hypothesis, which is not novel but has not been explored in the spatial domain, is that the thalamus supports inferential coding by enabling rapid and BD comparisons of incoming and stored information, with the place cells providing the (perceived or inferred) location to help other systems interpret *their* inputs, and those systems in turn feeding back their conclusions to the thalamus. Our planned experiments will try to investigate this hypothesis, which—if my previous form is anything to go by—is almost certainly wrong!

## 2 Reflections

I landed in the spatial coding field by accident, because the only behavioral neuroscience researchers within reach of my research‐curious medical‐student self, in 1980s New Zealand, just happened to be working on hippocampus. Back then I had no idea where the hippocampus is nor what it does, but was intrigued enough to sign up for an MSc with Graham Goddard, who had recently come from Dalhousie (see more on this in McNaughton 2025) to the Otago University psychology department to bring neuroscience and psychology together there. Graham had discovered, in the amygdala, the plasticity phenomenon known as kindling, and I was to work on that. Tragically, he drowned in a storm‐swollen river while hiking, just before I was to start.^5^ However his colleague Cliff Abraham took me under his wing, diverted me to LTP and shepherded me through my MSc and my first‐ever paper, on LTP maintenance (Jeffery et al. 1990). Through this work I learned about the research of Richard Morris (reviewed in Morris 2025), and wrote to him to ask if I could come to his lab in Edinburgh to do a PhD. It was there that Jim and I met: he was working in Brendan McGonigle's psychology lab next door to Richard's, trying to get robots to navigate (a still not‐well‐solved problem). I invited him to come next door and see my navigating rats, and thus began our long and fruitful collaboration (most notably producing three wonderful daughters).

All of which is to say, that serendipity has played a huge part in how both my research career and my life in general have unfolded. I feel very privileged to have stumbled by accident into one of the most exciting enterprises in neuroscience in the past century: the painstaking unraveling of a fundamentally important cognitive system in the brain—only the first system of hopefully many to be understood in such detail. The recent advent of modern recording, imaging and genetic methodologies has opened up new vistas of data that computational methods are rapidly evolving to exploit, and it is an exciting time to be a young researcher in this area. The field has progressed from, as it were, single case studies to epidemiology. These advances enable many new insights not previously attainable and it will be exciting to see what the next decades bring.

As with medicine, though, the single case studies remain important and I hope that a place for old‐fashioned single‐cell recording will be allowed to remain. Apart from anything else, there is nothing quite like the pleasure of working with a single neuron for an entire afternoon, doing psychological experiments on it as if it is a tiny animal, to try and understand what makes it tick. From a scientific standpoint, a close‐up view of small details can provide insights that huge data might obscure. Would O'Keefe have discovered place cells if he had 100,000 cells to analyze? Maybe, but maybe not. It was the intense focus on the behavior of each single cell that caused him to go “That's funny…”, and in so doing shift the field of biological psychology on its axis.

## Author Contributions

Kate J. Jeffery is the sole author.

## Conflicts of Interest

The author declares the following competing interests: K.J.J. is a non‐shareholding director of Axona Ltd., a company that makes and sells tetrode‐based electrophysiology products.

## Data Availability

The author has nothing to report.

## Endnotes

^1^
I did at least *mention* the theta skipping in my paper: something that eagle‐eyed Mark Brandon spotted years later and kindly referenced in his classic paper on the phenomenon (Brandon et al. 2013).

^2^
Not really—I'm just kidding. The Moser group's painstaking and elegant unraveling of this complex network has been wonderful to watch and I feel not the slightest bit of chagrin that I missed making this discovery myself.

^3^
They actually move a lot more freely than one might think, as I discovered 1 day when I looked out of the kitchen window of our new house to see the apple tree full of expertly scampering rats. Our Home Office inspector (from the UK regulatory body for animal experiments) later confirmed that the rat is as good a small mammal as any for 3D experiments.

^4^
This observation is an example of something that John used to call an “existence proof”—not publishable (except perhaps as a figure in a paper) but indisputable, and enough to start to build theories around. A nest of speed cells was eventually found in MEC by Emilio Kropff (Kropff et al. 2015); see also (Hinman et al. 2016).

^5^
See this obituary https://www.ilae.org/files/ilaeFarewell/GrahamGoddardObituary1939%2D1987.pdf. My friends and I were also caught in the same storm, further south, and I emerged from the mountains to learn the tragic news of Graham's demise from the newspaper in the Queenstown coffee shop where we had gone to regroup. I was devastated, and pretty sure that was the end of my research career, but fortunately Cliff came to the rescue.
